# The roles of growth regulation and appendage patterning genes in the morphogenesis of treehopper pronota

**DOI:** 10.1098/rspb.2021.2682

**Published:** 2022-06-08

**Authors:** Anna M. Kudla, Ximena Miranda, H. Frederik Nijhout

**Affiliations:** ^1^ Department of Biology, Duke University, Durham, NC 27708, USA; ^2^ Escuela de Biología, Universidad de Costa Rica, San José, Costa Rica

**Keywords:** membracidae, treehopper, *Entylia carinata*, pronotum, morphogenesis, growth

## Abstract

Treehoppers of the insect family Membracidae have evolved enlarged and elaborate pronotal structures, which is hypothesized to involve co-opted expression of genes that are shared with the wings. Here, we investigate the similarity between the pronotum and wings in relation to growth. Our study reveals that the ontogenetic allometry of the pronotum is similar to that of wings in Membracidae, but not the outgroup. Using transcriptomics, we identify genes related to translation and protein synthesis, which are mutually upregulated. These genes are implicated in the eIF2, eIF4/p70S6K and mTOR pathways, and have known roles in regulating cell growth and proliferation. We find that species-specific differential growth patterning of the pronotum begins as early as the third instar, which suggests that expression of appendage patterning genes occurs long before the metamorphic molt. We propose that a network related to growth and size determination is the more likely mechanism shared with wings. However, regulators upstream of the shared genes in pronotum and wings need to be elucidated to substantiate whether co-option has occurred. Finally, we believe it will be helpful to distinguish the mechanisms leading to pronotal size from those regulating pronotal shape as we make sense of this spectacular evolutionary innovation.

## 1. Introduction

**Figure 1.**
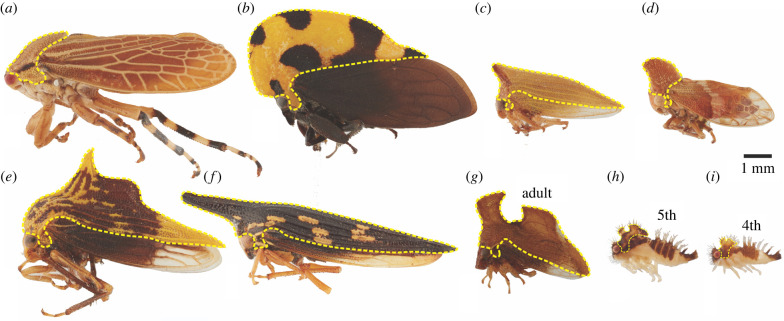
Treehopper species displaying variation in size and shape of the pronotum. (*a*–*c*, *e*–*g*) Adult treehopper species used in this study, including the outgroup (*a*) *Aetalion reticulatum* (Family: Aetalionidae), in which the pronotum, outlined in dashes (yellow in online version), is a small domed plate just behind the head, and five Membracidae, (*b*) *Membracis mexicana*, (*c*) *Metheisa lucillodes,* (*e*) *Ennya chrysura*, (*f*) *Polyglypta costata* and (*g*) *Entylia carinata*. The pronota of (*h*) 5th and (*i*) 4th instars of (*g*) *Ent. carinata* are much smaller than they are in the adult, but display the distal margin shape, an anterior horn and medial crest, characteristic of the species. (*d*) *Melizoderes variegata* from the family Melizoderidae, closely related to the Membracidae, does not have a posterior process and the exaggerated pronotal size. The pronotum, however, develops antereodorsally, diverging from the gently domed plate seen in *A. reticulatum* and most other hemipterans. (Online version in colour.)

The Membracidae, one of three families of treehoppers, are among the most morphologically diverse insect taxa (figure 1). The over 3300 species in this family have taken on forms to mimic thorns, twigs, seeds and fungi, as well as caterpillar frass, ants and wasps to name a few [1]. This incredible diversity arises from a single tissue, the pronotum (plural, pronota), which in most insects is a simple dorsal plate of the first thoracic segment [2]. Membracid pronota often extend to the tip of the abdomen and in some clades, completely cover the dorsal surface of the body so that even the wings are partially covered. Many clades also contain species with elaborate pronotal projections [3,4].

How the pronotum develops into a grossly enlarged structure with complex morphology is not understood. Recent work has shown that there is striking transcriptional similarity between the pronotum and the wing during the 5th instar. This work revealed a set of enriched gene ontology (GO) terms for 52 genes upregulated in the pronotum and wings relative to other body parts. They conclude that the transcriptional similarity between wings and pronotum is owing to shared and identifiable developmental processes. These results were used to expand the hypothesis that the pronotum co-opted the wing gene regulatory network during the evolution of its exaggerated morphologies in the membracids [5]. We suggest an examination of the genes that are transcriptionally shared between the pronotum and wings may shed light on developmental mechanisms that operate during morphogenesis of the membracid pronotum and clarify what processes may have been co-opted from the wings.

The metamorphic transition from the 5th instar to the adult of membracids (figure 1*g*,*h*) is associated with a massive increase in size of the pronotum and wings [6]. We hypothesize, therefore, that the pronotum and wings are transcriptionally similar owing to growth and size regulation in the final nymphal stage, the 5th instar. The comparable size change of the pronotum and wings is not found in most other insects. The membracid pronotum undergoes such tremendous growth that it develops as a complexly folded structure under the cuticle of the 5th instar before expanding during adult ecdysis [7]. Interestingly, in the 5th instar of another hemipteran, *Nilaparvata lugens* (Cicadellidae), the wings and genitalia are transcriptionally most similar to one another [5]. We suspect this transcriptional similarity is owing to a similarity in growth activity at this stage. To test this hypothesis, we investigate whether pathways related to protein synthesis, cell proliferation and growth, namely the insulin/mTOR signalling pathway, are upregulated in the pronotum and wings [8]. The insulin/mTOR signalling pathway has been well studied in insect systems and plays a major role in the regulation of organ size by controlling growth rate and duration [9–11]. Insulin is key for the coordination of whole-body growth such that the appropriate proportions of organs and appendages with overall body size are achieved [12,13].

Pronotal shape is probably owing to a variety of mechanisms including localized differential growth, changes in cell size and shape, cell migration and/or apoptosis much like the establishment of wing shape in butterflies [14–16]. We have preliminary evidence that the three-dimensional shape is established in earlier instars. We, therefore, hypothesize that the very attribute which makes the treehopper pronotum different from wings, its three-dimensional shape, is largely patterned and established along the dorsal surface prior to the final instar. No formal investigations into these earlier processes have been undertaken, hence it is unknown when and how growth is patterned in the pronotum. With these two hypotheses together, first, that transcriptional similarity is owing to shared mechanisms related to growth and size determination, and second, that pronotal dorsal shape is largely patterned and established before the 5th instar, we aim to make the distinction between the mechanisms that control pronotal growth and size, and the mechanisms that control pronotal shape.

In this study, we first investigate our assumptions related to pronotal size and shape. To test whether the changes in relative size of the pronotum and wing are comparable, we use ontogenetic allometries of pronotum and wing size relative to body size from five membracid species and compare those to *Aetalion reticulatum* (family, Aetalionidae), a closely related treehopper species that does not display the greatly enlarged pronotum. To examine the establishment of shape before the 5th instar, we use geometric morphometrics to compare pronotal shape in the 4th, 5th and adult stages in five membracid species. Finally, we explore the transcriptional similarity between the pronotum and wings of 5th instar *Entylia carinata*. To test for transcriptional similarities in patterning, we examine pronotal anterior-posterior (AP) axis patterning. Genes that are regionally expressed and pattern the AP axis of insect wings have been well documented [17–22]. We predict that if there is shared patterning between the pronotum and wings, these AP genes are likely to be differentially expressed in the anterior and posterior pronotum.

## 2. Methods

### (a) Ontogenetic allometry: size analysis

The sample (*n* = 174) consisted of six species, *Ent. carinata*, *Ennya chrysura*, *Polyglypta costata*, *Metheisa lucillodes*, *Membracis mexicana* and *A. reticulatum*. *Entylia carinata* were collected from a Duke colony, all other specimens from San José, Costa Rica (altitude 1200-1400 m) under permit SINAC-ACC-PI-R-018-2020. *Aetalion reticulatum* is from the treehopper family, Aetalionidae, which is either sister to or paraphyletic with the clade containing Membracidae [23]. *Aetalion reticulatum* served as the outgroup being from a closely related clade that does not display the derived enlarged and exaggerated pronotal characteristics present in most Membracidae. The 4th and 5th instars and the adults were used. For each species and stage, a minimum of five replicates were available. The surface area of the wing-pad and the lateral side of the pronotum were measured. As a proxy for body size, the head width was chosen. An Olympus SZX16 microscope with an Olympus DP71 camera and a Canon 5D Mark IV camera for larger individuals were used to photograph specimens. All measurements were performed in Fiji (v. 2.1). To obtain comparable units, the square roots of the wing and pronotum surface area measurements were taken. Head width, wing square root and pronotum square root were log transformed for use of linear allometric equation*, log y = α log x + log b*, to obtain the allometric coefficients of body size to wing and body size to pronotum across species.

### (b) Geometric morphometrics: shape analysis

The above sample was used for the ontogenetic allometry of shape, but without *A. reticulatum* and the addition of adult replicates for all membracids (*n* = 169). To quantify shape, the dorsal outline of 4th, 5th, and adults of all five species were digitized in lateral view, the orientation that contains species variation, and has been used as a taxonomic feature [24]. Two fixed landmarks were used at the most anterior and posterior points of the pronotum (electronic supplementary material, figure S1). Sixteen sliding semi-landmarks were placed along the dorsal margin. All specimens were photographed in lateral view as described above. Image digitization was done in tpsDIG2 and superimposition and analyses performed in RStudio (v. 1.2.5001).

### (c) Transcriptomics

#### (i) Rearing and RNA extraction

Day three 5th instar nymphs of *Ent. carinata* (figure 1*h*) were collected to analyse gene expression profiles. The forewings (T2 wings), the forelegs (T1 legs), the anterior pronotum, and the posterior pronotum (T1) were removed from each animal in a total of four specimens (*n* = 16) (electronic supplementary material, figure S1). All replicates were dissected immediately after anesthetization in CO_2_ and moved into TRIzol (Invitrogen). RNA was extracted and purified according to the RNA Mini Kit (Invitrogen) protocol with the DNase step. The quantity and quality of the RNA samples were determined using a Bioanalyzer 2100 with an Agilent RNA 6000 Pico chip. RNA samples were stored at −80°C until all replicates were ready for processing.

#### (ii) RNA-sequencing data and analysis

Library construction and sequencing were performed by the Sequencing and Genomic Technologies Core through the Duke Center for Genomic and Computational Biology (DCGCB). Libraries were prepared with the Clontech Ultra Low Input mRNA-seq (Illumina). NovaSeq 6000 S-Prime was used for sequencing and 50 bp paired-end reads were collected. Transcriptomic data preprocessing was performed through the Genomic Analysis and Bioinformatics Core (DCGCB). RNA-seq data were processed using the TrimGalore (v. 0.6.3) toolkit which employs Cutadapt to trim low-quality bases and Illumina sequencing adapters from the 3′ end of the reads [25]. Only reads that were 20 nucleotides or longer after trimming were kept for further analysis. Reads were mapped to the treehopper transcriptome (NCBI accession number GHWZ00000000) and quantified using Salmon (v. 1.3.0) [5,26]. Transcript-level abundance estimates from Salmon were converted into gene-level abundance estimates using the tximport package [27]. Only genes that had at least 10 reads in at least four libraries were used in subsequent analysis. Normalization and differential expression were carried out using the DESeq2 Bioconductor package (v. 1.16.1) with the R statistical programming environment (v. 3.2.2) [28,29]. Animal identity (ID) was used as a cofactor in each pairwise tissue comparison. A false discovery rate (FDR) was determined for all 13 367 genes tested using the method described in [30]. Differentially expressed (DE) data were merged in RStudio with *Ent. carinata* transcriptome annotations obtained from the Open Science Framework (OSF) [31]. Of the 13 367 genes obtained from sequencing, 3483 had Gene ID annotations and clear orthology. The remaining genes had only GO term annotations or no annotation. These 3483 genes were used to identify mutually upregulated genes in the pronotum and wings and as input for pathway analysis, which requires gene IDs (see the electronic supplementary material, methods and table S2 for more details).

#### (iii) Comparisons of shared gene expression with pronotum

Two subsets of gene expression data were generated from the Full Gene ID subset (3483 genes) to identify (i) genes that were mutually differentially expressed (DE) in the pronotum (anterior and posterior) and wings compared to the legs, and (ii) genes that were mutually DE in the pronotum and legs compared to the wings. To obtain these two datasets, DE genes across all pairwise comparisons were subset with an FDR of less than 0.05. Venn diagrams generated in RStudio with the *venn* package were used to identify the number of DE genes that were shared between pronotum and wings relative to legs, and pronotum and legs relative to wings.

#### (iv) Pathway analysis

Pathway enrichment analysis was performed to further investigate similarities and differences across pairwise comparisons of DE data for the four tissue types. The analysis was performed with QIAGEN ingenuity pathway analysis (IPA) [32]. IPA uses a powerful database with numerous canonical pathways and can hypothesize genetic correlations that cannot be predicted with other pathway analysis tools. However, IPA takes inputs from human or mouse genes only, and thus, *Mus musculus* orthologues were used. Three per cent of the *Ent. carinata* transcriptome with gene name annotations contained human and mouse genes. The remaining orthologues were identified with Ensembl (v. 104). Of the 3484 annotated genes, 3134 had orthologues to *Mus musculus*. For IPA, the non-DE genes were used as background. Pathways with a *p*-value < 0.05 were considered significant after Bonferroni correction. Additional pathway analyses were performed using the Database for Annotation, Visualization and Integrated Discovery (DAVID) to confirm IPA results (electronic supplementary material, analyses and table S1) [33,34].

### (d) Anterior-posterior differences

To investigate whether the anterior and posterior regions of the pronotum were similarly defined by the genes patterning the AP axis of *Drosophila melanogaster* wings, genes that characterize anterior and posterior identity, respectively, were identified through a literature search. Nine total genes, five anterior and four posterior were selected for their unique expression in one of the two regions (electronic supplementary material, figure S3). GO enrichment analyses were implemented in DAVID to identify gene groups with shared developmental processes. For this analysis, the AP pairwise comparison dataset was used. Genes that were upregulated in the anterior relative to the posterior (*p*-adjusted < 0.05) were input into DAVID for anterior GO enrichment and vice versa for the posterior with the *Drosophila melanogaster* genome as background. GO terms with *p*-adjusted < 0.05 were taken into consideration for analyses.

## 3. Results

### (a) Ontogenetic allometry: the relative growth of pronota and wings

**Figure 2.**
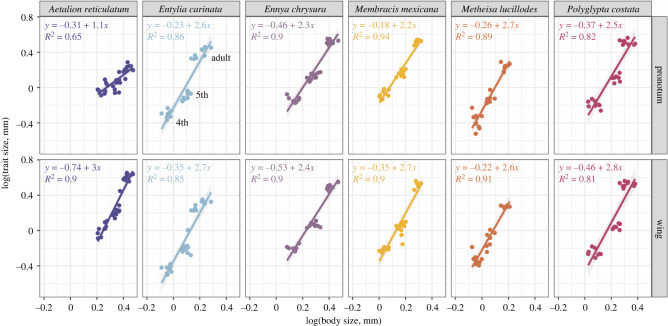
Ontogenetic allometry of pronotum and wing size relative to body size across six morphologically disparate treehopper species. The scaling relationships of the membracid pronotum and wing relative to body size in the 4th, 5th and adult stages, are noticeably similar compared to that of the outgroup, *Aetalion reticulatum*. There are large changes in the relative sizes of the pronotum in all membracids, with slopes (allometric coefficients) >2. These were similar to the relative size changes of the wing, with slopes >2. (Online version in colour.)

We found the scaling relationship (allometric coefficient or slope) of the wing and the pronotum to be similar in all membracids (pronotum and wing slope greater than 2) but not in the outgroup, *A. reticulatum* (figure 2). The scaling of the pronotum in *A. reticulatum* (slope = 1.1) was lower than the scaling of the wings (slope = 3.0). To validate that the similar allometric coefficients were unique to the pronotum and wings of membracids, we performed a check by measuring the length of *Ent. carinata* hindleg femurs (allometric coefficient = 1.6). We examined the untransformed surface (mm^2^) area data to confirm our results were not the product of data transformation (electronic supplementary material, figure S2). This revealed a similar pattern in which the pronotum surface area and wing surface area scaled close to a 1 to 1 ratio in Membracidae, but not in *A. reticulatum* (slope = 0.11).

### (b) Geometric morphometrics: the ontogeny of shape

**Figure 3.**
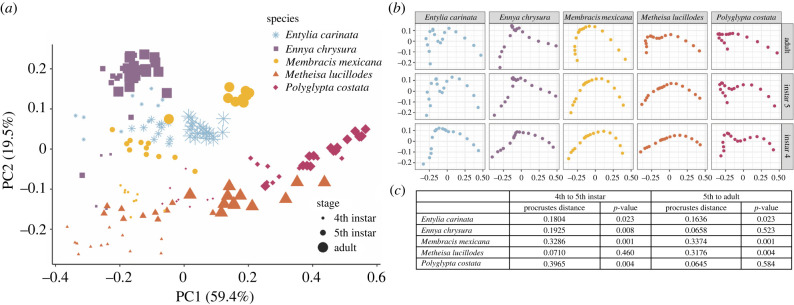
Dorsal surface shape comparisons of the pronotal tissue in 4th instars, 5th instars, and adults across five membracid species. (*a*) Morphospace displaying PC1 and PC2 emphasize that there is little to no overlap in shapes across species, within a developmental stage (size of symbol). (*b*) Qualitative shape variation across developmental stages and species shows differences emerge early and continue to diverge with progressive stages. (*c*) Results of Procrustes ANOVA show a significant difference in shape between the 4th and the 5th, which for some species is larger than the shape difference between the 5th and the adult. There are still significant shape differences between the 5th and the adult for several species, but this was not a consistently observed pattern across all species. (Online version in colour.)

Our data show that some of the attributes that contribute to pronotal form are already present in the 4th and 5th instars. The morphospace generated from principal components analysis (PCA) showed elements of shape that corresponded to ontogeny along PC1, and elements that corresponded to species differences along PC2 (figure 3*a*). PC1 accounted for 59.4% of the shape variation and described the elongation along the AP axis. PC2 accounted for 19.5% of shape variation and corresponded to distal outgrowths of the pronotum. The mean shapes of pronota across species and at different developmental stages showed qualitative differences in overall shape changes (figure 3*b*). We found that adult morphological features emerged before the 5th (final) instar.

Our geometric morphometric analysis showed that in some species there were aspects of shape that changed between the 5th instar and the adult (figure 3*c*). Three of the five species showed significant differences in shape between the 5th instar and the adult based on Procrustes distance, a quantification of the degree of shape difference (figure 3*c*). In *Met. lucillodes*, there was a noticeable emergence of an anterior bump in the adult. The shape changes in *Ent. carinata* and *Mem. mexicana* were more subtle and mostly owing to forward growth of the anterior process that occurred from the 5th to the adult. However, the characteristic crest of *Mem. mexicana* and the anterior and medial crests characteristic of *Ent. carinata* were already present in the 5th instar. In all species except *Met. lucillodes*, several features of the adult pronotum were already established in the 4th instar (figure 3*a*).

### (c) RNAseq

We used RNA sequencing to examine shared gene expression patterns between the pronotum and the wings after these structures were shown to be transcriptionally similar in 5th instar *Ent. carinata* [5]. To specifically investigate whether the similarity between the pronotum and wings was associated with genes related to the control of growth, we compared the anterior and posterior pronotum (T1), to the forewings (T2), and to the forelegs (T1). Of the 3483 genes with Gene IDs, we found 200 to be DE between the anterior pronotum and the legs, 529 between the posterior pronotum and the legs, and 493 between the wings and the legs (*p*-adjusted < 0.05). Pairwise comparison between anterior pronotum and wings showed 191 DE genes, and pairwise comparison between the posterior pronotum and wings showed 123 DE genes.

#### (i) Shared differential gene expression

**Figure 4.**
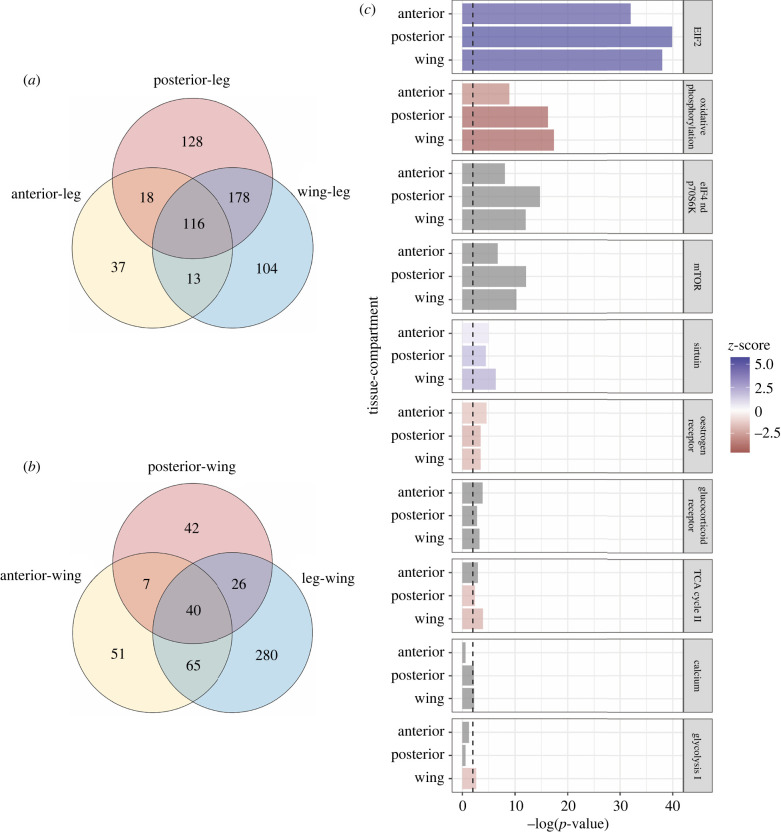
Results from comparative transcriptomic analyses of the pronotum (anterior and posterior), wings and legs: overlapping significantly differentially expressed (DE) genes shared in the anterior and posterior pronotum with (*a*) wings and (*b*) legs respectively (FDR < 0.05) are shown in the Venn diagram. (*a*) There were 116 mutually significantly DE genes in the wings, anterior and posterior pronotal tissues compared to legs and (*b*) 40 mutually significantly DE genes in the legs, anterior, and posterior pronotal tissues compared to legs (see the electronic supplementary material, tables S3 and S4 for full list of genes). (*c*) The top significantly enriched canonical pathways in wings from ingenuity pathway analysis (IPA) show several pathways mutually enriched in the pronotum (anterior and posterior) and wings. Positive *z*-scores (purple in online version) indicate an upregulation of the pathway relative to legs. Negative *z*-scores (red in online version) indicate a downregulation of the pathway relative to legs. Four significantly enriched canonical pathways did not have enough data to determine whether the pathway was upregulated or downregulated in the pronotum and wings relative to legs (grey in online version). (Online version in colour.)

The anterior and posterior pronota and wings had more overlap of DE genes (figure 4*a*, 116 genes) with the legs as the comparison group, than pronota and legs had with wings as the comparison group (figure 4*b*, 40 genes). Thus, pronota and wings shared more DE genes than pronota and legs. We expected this, given previous findings of pronotal-wing similarity. We used the pronota comparison with legs to confirm that the shared genes between the pronota and the wings were unique. Most of the shared upregulated genes were related to ribosomal proteins (53 genes), elongation factors (four genes), and molecular machinery for protein synthesis (three genes) (electronic supplementary material, table S3). *Spalt* and *invected*, well characterized in developing wings, were mutually upregulated in the pronotum and wings, and *engrailed* and *vestigial* were mutually upregulated in the posterior pronotum and wings. A different suite of genes emerged in the comparison of pronota and legs (electronic supplementary material, table S4). Unsurprisingly, we found *Sex combs reduced* (*Scr*), a homeotic gene responsible for T1 identity to be shared between the pronota and legs, which are both part of the T1 region.

#### (ii) Ingenuity pathway analysis canonical pathways

IPA predicted the eukaryotic initiation factor 2 (eIF2) signalling pathway to be significantly upregulated in the wings and anterior and posterior pronotum relative to the legs (figure 4*c*). eIF2 plays a major role in the regulation of translation [35,36]. IPA also showed the sirtuin signalling pathway to be significantly enriched in the pronota and wings relative to the legs, but only significantly upregulated in the posterior pronotum and wings. Sirtuin 1 is involved in the activation of Akt in the insulin signalling pathway [37]. We observed a few pathways downregulated in the pronotum and wings relative to the legs including oxidative phosphorylation and oestrogen receptor signalling. We found the mTOR signalling pathway and regulation of eIF4 and p70S6K signalling, important for the regulation of translation, to be significantly enriched, but there was not enough information to determine the directionality of regulation. However, these pathways are indirectly related to EIF2 signalling since both mTOR and p70S6K signalling pathways are upstream of protein synthesis [38,39].

#### (iii) Anterior and posterior pronotum patterning

To examine the possibility that the pronotum shared genes that are known to pattern the wing, we first separated the anterior and posterior portions of the pronotum. We wanted to identify whether differences existed with respect to AP axis patterning genes, which are well studied in *D. melanogaster* wings [20,21,40,41]. The pairwise DE data showed that there were 295 DE genes between the anterior and posterior pronotal regions (electronic supplementary material, figure S3). Based on our GO term enrichment analysis, many of the genes upregulated in the anterior relative to the posterior were related to muscle development (*Mhc*, *ScgDelta*, *Actn*, *bt,* *sls*, *wupA*, *sr*, *nau*, *Tm1*, *unc-5*) and mesoderm development (*Msp300*, *Prm*, *Zasp66*, *twi*). We found the genes upregulated in the posterior to be related to translation (ribosomal proteins). Two of the nine AP axis patterning genes we selected, *engrailed* and *invected*, were DE (electronic supplementary material, figure S3). Both genes are transcription factors that are co-regulated throughout *D. melanogaster* development and are necessary to establish posterior compartment identity in wings, legs and the embryo [21,42–44].

## 4. Discussion

### (a) Mechanisms related to growth

In this study, we compared size and shape ontogenies of pronota and wings in six species of treehoppers and we explored the transcriptional similarities and differences between two regions of the pronotum, the wings, and foreleg of *Ent. carinata*. We found the relative growth in the pronotum and wings to be similar from the 4th to the 5th instar and to the adult. This was not the case for the outgroup treehopper, *A. reticulatum*, in which the relative growth of the pronotum was much less than that of the wings. These results suggest that the membracid pronotum became more similar in size to the wing, diverging from the ancestral pronotal relative size over the course of evolution. The similarity between the relative size changes of the pronotum and wings to achieve similar sizes makes growth itself an important attribute that must be considered when explaining the transcriptional similarity.

The transcriptomic results suggest that molecular factors related to growth at the 5th instar stage of development is a major contributor to the transcriptional similarity of the pronotum and wings. By comparing the pronotum and wings to the T1 legs, we identified a suite of genes important for translation regulation. Pathway analysis revealed that the eIF2 pathway was highly enriched and upregulated in the pronotum and wings. eIF2 is a necessary component of translational machinery, protein synthesis and cell proliferation [16,35,45–48]. We, however, expected to find similar molecular factors related to the regulation of growth between pronotum and wings, namely, components of the insulin/mTOR signalling pathways. The mTOR signalling pathway was enriched in the pronotum and wings relative to the legs, but there were not enough data to determine the directionality. Instead, our results show transcriptional similarities downstream of growth regulation pathways with an indication that the insulin/mTOR pathway may have a role in the expression of the ribosomal proteins we observed. Given that eIF2 was highly upregulated in the pronotum and wings, we tentatively assume that mTOR was also activated in these structures and it has been suggested these two pathways are linked [38,49,50]. Namely, they may share in the regulation by protein kinase B (PKB) or AKT, a key component of the insulin signalling pathway [51–53].

Finally, we analysed differences in the anterior and posterior portions of the pronotum to test whether axis patterning genes were regionally expressed as one potential alternative to transcriptional similarity relating to growth mechanisms. We found *engrailed/invected* to be significantly upregulated in the posterior pronotum, but no other patterning genes were. *Engrailed/invected* are frequently found to be constitutively expressed in the posterior compartment of developing tissues [20]. Other genes related to AP axis patterning might be expressed in earlier instars that could lead to development of the distinct anterior bump and medial hump traits of *Ent. carinata*. Our finding also suggests that this particular component of what is often called the wing gene regulatory network is not expressed in the pronotum at this time of the 5th instar [54].

Our findings on transcriptional similarity differ from those in the previous work on treehopper transcriptomics [5]. We found that genes related to ribosomes and protein synthesis were shared between the pronotum and wings. This difference may be owing to the time at which the specimens were sampled, and the structures chosen for comparison. We chose a timepoint to maximize the possibility of observing tissue patterning mechanisms, just prior to cell proliferation. Our analysis had strict requirements, genes with known orthology, which led to a reduced data subset. Currently, a completely annotated transcriptome is unavailable for any membracid. With the completion of a transcriptome, future investigations will shed further light on the causes and consequences of transcriptional similarity between pronotum and appendages in membracids.

### (b) Pronotum evolution: co-option or convergence?

Although genes expressed in wings are also expressed in the pronotum, a more nuanced interpretation of the significance of this association is required [55,56]. We suggest that two things must be considered when investigating questions related to treehopper pronotal development and evolution: (i) the genes frequently studied in wings, such as *wingless, distal-less, engrailed, hedgehog, spalt, decapentaplegic, apterous, vestigial*, are also expressed in, and responsible for the patterning of, other appendages including legs, antennae, mouthparts and genitalia, and also play critical roles earlier, in embryonic development [2,21,57,58]; and (ii) our observations show that many general features of the adult pronotum are already evident in the 4th instar (figure 3), which suggests that critical transcriptional patterning of pronotal differentiation occurs before the 5th instar. Species-specific morphologies are progressively patterned during the nymphal stages and comparative work on the timing and the location of gene expression in earlier instars will be key for understanding the developmental and evolutionary changes that lead to morphological variation.

This leads to the question of whether the wing gene network had been co-opted in the development of the exaggerated pronotum of the Membracidae. Does the transcriptional similarity between the pronotum and the wings still point to the wing-gene network, considering our findings that translation-related genes appear to dominate among the differentially expressed genes in the pronotum? The challenge in proving co-option of a network rather than the re-use of single genes is the need to demonstrate that the genes from the co-opted network are fully expressed in the novel structure and share both topological and regulatory components with the network that was co-opted [59,60]. This is a challenge further exacerbated by the fact that the genes which are well studied in wing development are expressed in many other developing structures throughout ontogeny and morphogenesis. Therefore, it is not a simple task to determine from which antecedent structure a new morphological feature was co-opting. Ultimately, all the genes in the wing network and in the pronotum have complex expression patterns and regulatory functions in the embryo, and the full pathway by which these genes acquired novel functionalities in late-developing appendages and other structures is yet unknown.

Understanding the regulation of protein synthesis and localized growth will be essential to recognize the commonalities and differences between the mechanisms that pattern the wing and the pronotum. Components of the insulin/mTOR signalling pathway are probably shared by wings and pronota and may be convergently deployed to manage the disproportional growth of these two structures. It is possible that co-option of regulatory factors upstream of insulin/mTOR has occurred, and it is possible that those upstream factors also regulate the expression of genes involved in wing development. For example, a handful of genes were found to be uniquely enriched in the membracid pronotum but not the leafhopper pronotum [5]. Interestingly, these genes have varying functions in different tissues. In wings they have a role in the regulation of growth (*apterous, four-jointed, serum response factor*), planar polarity, cell size, epithelial growth and epithelial repair (*grainy head, miniature, four-jointed*), cell polarity and tissue symmetry (*frizzled*) and tracheal development (*serum response factor*) [61–71]. When deployed in a new cellular and molecular context, there is no guarantee that these genes will preserve their ancestral function. An exploration of questions pertaining to the control of growth, those related to relative growth and those to localized growth patterning, will be essential for understanding the development and evolution of pronotal shape diversity and may also give more insights into the mechanisms that lead to morphological diversification in general.

## Data Availability

Data and code used to analyse and generate figures for allometry, geometric morphometrics and RNAseq work are available through the Dryad Digital Repository: https://doi.org/10.5061/dryad.8pk0p2npg [72]. Raw sequence files are found in NCBI BioProject no. PRJNA817220 with accession nos. SRR18360271 through SRR18360286. An electronic supplementary material file with additional methods, analyses, figures, and tables has been included to further support this study [73].

## References

[1] Wood TK. 1993 Diversity in the New World Membracidae. Annu. Rev. Entomol. **38**, 409-433. (10.1146/annurev.en.38.010193.002205)

[2] Calleja M, Renaud O, Usui K, Pistillo D, Morata G, Simpson P. 2002 How to pattern an epithelium: lessons from achaete-scute regulation on the notum of *Drosophila*. Gene **292**, 1-12. (10.1016/S0378-1119(02)00628-5) 12119094

[3] Godoy C, Miranda X, Nishida K. 2006 Treehoppers of Tropical America/Membrácidos de la América Tropical, 1st edn. Santo Domingo, Heredia, Costa Rica. Instituto Nacional de Biodiversidad. See https://www.nhbs.com/treehoppers-of-tropical-america-membracidos-de-la-america-tropical-book.

[4] Evangelista O, Sakakibara AM, Cryan JR, Urban JM. 2017 A phylogeny of the treehopper subfamily Heteronotinae reveals convergent pronotal traits (Hemiptera: Auchenorrhyncha: Membracidae). Syst. Entomol. **42**, 410-428. (10.1111/syen.12221)

[5] Fisher CR, Wegrzyn JL, Jockusch EL. 2020 Co-option of wing-patterning genes underlies the evolution of the treehopper helmet. Nat. Ecol. Evol. **4**, 250-260. (10.1038/s41559-019-1054-4) 31819237

[6] Stegmann UE. 1998 An exaggerated trait in insects: the prothoracic skeleton of *Stictocephala bisonia* (Homoptera: Membracidae). J. Morphol. **238**, 157-178. (10.1002/(SICI)1097-4687(199811)238:2<157::AID-JMOR3>3.0.CO;2-H) 29852717

[7] Adachi H, Matsuda K, Nishida K, Hanson P, Kondo S, Gotoh H. 2020 Structure and development of the complex helmet of treehoppers (Insecta: Hemiptera: Membracidae). Zool. Lett. **6**, 3. (10.1186/s40851-020-00155-7) PMC7041272 32123574

[8] Nijhout HF, Callier V. 2013 A new mathematical approach for qualitative modeling of the insulin-TOR-MAPK network. Front. Physiol. **4**, 245. (10.3389/fphys.2013.00245) 24062690 PMC3771213

[9] Mirth CK, Anthony Frankino W, Shingleton AW. 2016 Allometry and size control: what can studies of body size regulation teach us about the evolution of morphological scaling relationships? Curr. Opin. Insect Sci. **13**, 93-98. (10.1016/j.cois.2016.02.010) 27436558

[10] Vea IM, Shingleton AW. 2021 Network-regulated organ allometry: the developmental regulation of morphological scaling. WIREs Dev. Biol. **10**, e391. (10.1002/wdev.391) 32567243

[11] McKenna KZ, Tao D, Nijhout HF. 2019 Exploring the role of insulin signaling in relative growth: a case study on wing-body scaling in Lepidoptera. Integr. Comp. Biol. **59**, 1324-1337. (10.1093/icb/icz080) 31141129

[12] Texada MJ, Koyama T, Rewitz K. 2020 Regulation of body size and growth control. Genetics **216**, 269-313. (10.1534/genetics.120.303095) 33023929 PMC7536854

[13] Shingleton AW, Frankino WA, Flatt T, Nijhout HF, Emlen DJ. 2007 Size and shape: the developmental regulation of static allometry in insects. Bioessays **29**, 536-548. (10.1002/bies.20584) 17508394

[14] Nijhout HF, Cinderella M, Grunert LW. 2014 The development of wing shape in Lepidoptera: mitotic density, not orientation, is the primary determinant of shape. Evol. Dev. **16**, 68-77. (10.1111/ede.12065) 24617986

[15] McKenna KZ, Nijhout HF. 2021 The development of shape. Modular control of growth in the lepidopteran forewing. J. Exp. Zool. B: Mol. Dev. Evol. **338**, 170-180. (10.1002/jez.b.23101) 34710273

[16] Emlen DJ, Szafran Q, Corley LS, Dworkin I. 2006 Insulin signaling and limb-patterning: candidate pathways for the origin and evolutionary diversification of beetle ‘horns’. Heredity **97**, 179-191. (10.1038/sj.hdy.6800868) 16850039

[17] Schwartz C, Locke J, Nishida C, Kornberg TB. 1995 Analysis of cubitus interruptus regulation in *Drosophila* embryos and imaginal disks. Development **121**, 1625-1635. (10.1242/dev.121.6.1625) 7600980

[18] Hidalgo A. 1994 Three distinct roles for the engrailed gene in *Drosophila* wing development. Curr. Biol. **4**, 1087-1098. (10.1016/S0960-9822(00)00247-5) 7704572

[19] Ibrahim DM, Biehs B, Kornberg TB, Klebes A. 2013 Microarray comparison of anterior and posterior *Drosophila* wing imaginal disc cells identifies novel wing genes. G3 Genes|Genomes|Genetics **3**, 1353-1362. (10.1534/g3.113.006569) 23749451 PMC3737175

[20] Basler K, Struhl G. 1994 Compartment boundaries and the control of *Drosophila* limb pattern by hedgehog protein. Nature **368**, 208-214. (10.1038/368208a0) 8145818

[21] Morata G, Lawrence PA. 1975 Control of compartment development by the engrailed gene in *Drosophila*. Nature **255**, 614-617. (10.1038/255614a0) 1134551

[22] Campbell G, Weaver T, Tomlinson A. 1993 Axis specification in the developing *Drosophila* appendage: the role of wingless, decapentaplegic, and the homeobox gene aristaless. Cell **74**, 1113-1123. (10.1016/0092-8674(93)90732-6) 8104704

[23] Dietrich CH, Allen JM, Lemmon AR, Lemmon EM, Takiya DM, Evangelista O, Walden KKO, Grady PGS, Johnson KP. 2017 Anchored hybrid enrichment-based phylogenomics of leafhoppers and treehoppers (Hemiptera: Cicadomorpha: Membracoidea). Insect Syst. Divers. **1**, 57-72. (10.1093/isd/ixx003)

[24] Mckamey S, Deitz L. 1996 Generic revision of the New World tribe Hoplophorionini (Hemiptera: Membracidae: Membracinae). Syst. Entomol. **21**, 295-342. (10.1111/j.1365-3113.1996.tb00602.x)

[25] Martin M. 2011 Cutadapt removes adapter sequences from high-throughput sequencing reads. EMBnet J. **17**, 10-12. (10.14806/ej.17.1.200)

[26] Patro R, Duggal G, Love MI, Irizarry RA, Kingsford C. 2017 Salmon provides fast and bias-aware quantification of transcript expression. Nat. Methods **14**, 417-419. (10.1038/nmeth.4197) 28263959 PMC5600148

[27] Soneson C, Love MI, Robinson MD. 2015 Differential analyses for RNA-seq: transcript-level estimates improve gene-level inferences. F1000Res **4**, 1521. (10.12688/f1000research.7563.2) 26925227 PMC4712774

[28] Love MI, Huber W, Anders S. 2014 Moderated estimation of fold change and dispersion for RNA-seq data with DESeq2. Genome Biol. **15**, 550. (10.1186/s13059-014-0550-8) 25516281 PMC4302049

[29] Huber AB et al. 2005 Distinct roles for secreted semaphorin signaling in spinal motor axon guidance. Neuron **48**, 949-964. (10.1016/j.neuron.2005.12.003) 16364899

[30] Benjamini Y, Hochberg Y. 1995 Controlling the false discovery rate: a practical and powerful approach to multiple testing. J. R. Stat. Soc.: B (Methodol.) **57**, 289-300. (10.1111/j.2517-6161.1995.tb02031.x)

[31] Fisher C. 2019 *Entylia carinata* refined transcriptome assembly and annotations. See osf.io/zs5hy.

[32] Krämer A, Green J, Pollard Jr J, Tugendreich S. 2014 Causal analysis approaches in ingenuity pathway analysis. Bioinformatics **30**, 523-530. (10.1093/bioinformatics/btt703) 24336805 PMC3928520

[33] Huang DW, Sherman BT, Lempicki RA. 2009 Systematic and integrative analysis of large gene lists using DAVID bioinformatics resources. Nat. Protoc. **4**, 44-57. (10.1038/nprot.2008.211) 19131956

[34] Huang DW, Sherman BT, Lempicki RA. 2009 Bioinformatics enrichment tools: paths toward the comprehensive functional analysis of large gene lists. Nucleic Acids Res. **37**, 1-13. (10.1093/nar/gkn923) 19033363 PMC2615629

[35] Kimball SR. 1999 Eukaryotic initiation factor eIF2. Int. J. Biochem. Cell Biol. **31**, 25-29. (10.1016/S1357-2725(98)00128-9) 10216940

[36] Pavitt GD, Ramaiah KVA, Kimball SR, Hinnebusch AG. 1998 eIF2 independently binds two distinct eIF2B subcomplexes that catalyze and regulate guanine–nucleotide exchange. Genes Dev. **12**, 514-526. (10.1101/gad.12.4.514) 9472020 PMC316533

[37] Liang F, Kume S, Koya D. 2009 SIRT1 and insulin resistance. Nat. Rev. Endocrinol. **5**, 367-374. (10.1038/nrendo.2009.101) 19455179

[38] Wengrod J, Wang D, Weiss S, Zhong H, Osman I, Gardner LB. 2015 Phosphorylation of eIF2*α* triggered by mTORC1 inhibition and PP6C activation is required for autophagy and is aberrant in PP6C-mutated melanoma. Sci. Signal. **8**, ra27. (10.1126/scisignal.aaa0899) 25759478 PMC4580977

[39] Welsh GI, Miller CM, Loughlin AJ, Price NT, Proud CG. 1998 Regulation of eukaryotic initiation factor eIF2B: glycogen synthase kinase-3 phosphorylates a conserved serine which undergoes dephosphorylation in response to insulin. FEBS Lett. **421**, 125-130. (10.1016/s0014-5793(97)01548-2) 9468292

[40] Williams JA, Paddock SW, Vorwerk K, Carroll SB. 1994 Organization of wing formation and induction of a wing-patterning gene at the dorsal/ventral compartment boundary. Nature **368**, 299-305. (10.1038/368299a0) 8127364

[41] Abbasi R, Marcus JM. 2017 A new A-P compartment boundary and organizer in holometabolous insect wings. Sci. Rep. **7**, 1-11. (10.1038/s41598-017-16553-5) 29180689 PMC5704014

[42] Hatini V, DiNardo S. 2001 Divide and conquer: pattern formation in *Drosophila* embryonic epidermis. Trends Genet. **17**, 574-579. (10.1016/S0168-9525(01)02448-9) 11585663

[43] Simmonds AJ, Brook WJ, Cohen SM, Bell JB. 1995 Distinguishable functions for engrailed and invected in anterior–posterior patterning in the *Drosophila* wing. Nature **376**, 424-427. (10.1038/376424a0) 7630417

[44] Cheng Y, Brunner AL, Kremer S, DeVido SK, Stefaniuk CM, Kassis JA. 2014 Co-regulation of invected and engrailed by a complex array of regulatory sequences in *Drosophila*. Dev. Biol. **395**, 131-143. (10.1016/j.ydbio.2014.08.021) 25172431 PMC4189978

[45] Williams DD, Pavitt GD, Proud CG. 2001 Characterization of the initiation factor eIF2B and its regulation in *Drosophila melanogaster*. J. Biol. Chem. **276**, 3733-3742. (10.1074/jbc.M008041200) 11060303

[46] Johnston LA, Gallant P. 2002 Control of growth and organ size in *Drosophila*. Bioessays **24**, 54-64. (10.1002/bies.10021) 11782950

[47] Rios-Fuller TJ et al. 2020 Translation regulation by eIF2*α* phosphorylation and mTORC1 signaling pathways in non-communicable diseases (NCDs). Int. J. Mol. Sci. **21**, E5301. (10.3390/ijms21155301) PMC7432514 32722591

[48] Hao P, Yu J, Ward R, Liu Y, Hao Q, An S, Xu T. 2020 Eukaryotic translation initiation factors as promising targets in cancer therapy. Cell Commun. Signal. **18**, 175. (10.1186/s12964-020-00607-9) 33148274 PMC7640403

[49] Paccalin M, Barc SP, Paquet C, Pluchon C, Bilan AR, Gil R, Hugon J. 2006 P3–096: kinases mTOR and PKR controlling translation in Alzheimer's disease potential biomarkers? Alzheimer's Dementia **2**, S401. (10.1016/j.jalz.2006.05.1363)

[50] Rosario FJ, Powell TL, Gupta MB, Cox L, Jansson T. 2020 mTORC1 transcriptional regulation of ribosome subunits, protein synthesis, and molecular transport in primary human trophoblast cells. Front. Cell Dev. Biol. **8**, 583801. (10.3389/fcell.2020.583801) 33324640 PMC7726231

[51] Britton JS, Lockwood WK, Li L, Cohen SM, Edgar BA. 2002 *Drosophila*'s insulin/PI3-kinase pathway coordinates cellular metabolism with nutritional conditions. Dev. Cell **2**, 239-249. (10.1016/S1534-5807(02)00117-X) 11832249

[52] Lin X, Smagghe G. 2019 Roles of the insulin signaling pathway in insect development and organ growth. Peptides **122**, 169923. (10.1016/j.peptides.2018.02.001) 29458057

[53] Puig O, Tjian R. 2005 Transcriptional feedback control of insulin receptor by dFOXO/FOXO1. Genes Dev. **19**, 2435-2446. (10.1101/gad.1340505) 16230533 PMC1257398

[54] Abouheif E, Wray GA. 2002 Evolution of the gene network underlying wing polyphenism in ants. Science **297**, 249-252. (10.1126/science.1071468) 12114626

[55] Fisher CR, Kratovil JD, Angelini DR, Jockusch EL. 2021 Out from under the wing: reconceptualizing the insect wing gene regulatory network as a versatile, general module for body-wall lobes in arthropods. Proc. R. Soc. B **288**, 20211808. (10.1098/rspb.2021.1808) PMC8692954 34933597

[56] DiFrisco J, Wagner GP, Love AC. 2022 Reframing research on evolutionary novelty and co-option: character identity mechanisms versus deep homology. Semin. Cell Dev. Biol. **126**. (10.1016/j.semcdb.2022.03.030) 35400563

[57] Dong PDS, Dicks JS, Panganiban G. 2002 Distal-less and homothorax regulate multiple targets to pattern the *Drosophila* antenna. Development **129**, 1967-1974. (10.1242/dev.129.8.1967) 11934862

[58] Estella C, Voutev R, Mann RS. 2012 A dynamic network of morphogens and transcription factors patterns the fly leg. Curr. Top. Dev. Biol. **98**, 173-198. (10.1016/B978-0-12-386499-4.00007-0) 22305163 PMC3918458

[59] Glassford WJ, Johnson WC, Dall NR, Smith SJ, Liu Y, Boll W, Noll M, Rebeiz M. 2015 Co-option of an ancestral hox-regulated network underlies a recently evolved morphological novelty. Dev. Cell **34**, 520-531. (10.1016/j.devcel.2015.08.005) 26343453 PMC4573913

[60] McKenna KZ, Wagner GP, Cooper KL. 2021 Chapter One: a developmental perspective of homology and evolutionary novelty. In Current topics in developmental biology (ed. SF Gilbert), pp. 1-38. San Diego, CA: Academic Press. 10.1016/bs.ctdb.2020.12.001 33602485

[61] Newby LM, White L, DiBartolomeis SM, Walker BJ, Dowse HB, Ringo JM, Khuda N, Jackson FR. 1991 Mutational analysis of the *Drosophila* miniature-dusky (m-dy) locus: effects on cell size and circadian rhythms. Genetics **128**, 571-582. (10.1093/genetics/128.3.571) 1908397 PMC1204531

[62] Villano JL, Katz FN. 1995 four-jointed is required for intermediate growth in the proximal-distal axis in *Drosophila*. Development **121**, 2767-2777. (10.1242/dev.121.9.2767) 7555705

[63] Montagne J, Groppe J, Guillemin K, Krasnow MA, Gehring WJ, Affolter M. 1996 The *Drosophila* serum response factor gene is required for the formation of intervein tissue of the wing and is allelic to blistered. Development **122**, 2589-2597. (10.1242/dev.122.9.2589) 8787734

[64] Zeidler MP, Perrimon N, Strutt DI. 2000 Multiple roles for four-jointed in planar polarity and limb patterning. Dev. Biol. **228**, 181-196. (10.1006/dbio.2000.9940) 11112323

[65] Strutt DI. 2001 Asymmetric localization of frizzled and the establishment of cell polarity in the *Drosophila* wing. Mol. Cell **7**, 367-375. (10.1016/s1097-2765(01)00184-8) 11239465

[66] Han C, Belenkaya TY, Wang B, Lin X. 2004 *Drosophila glypicans* control the cell-to-cell movement of Hedgehog by a dynamin-independent process. Development **131**, 601-611. (10.1242/dev.00958) 14729575

[67] Mace KA, Pearson JC, McGinnis W. 2005 An epidermal barrier wound repair pathway in *Drosophila* is mediated by grainy head. Science **308**, 381-385. (10.1126/science.1107573) 15831751

[68] Seifert JRK, Mlodzik M. 2007 Frizzled/PCP signalling: a conserved mechanism regulating cell polarity and directed motility. Nat. Rev. Genet. **8**, 126-138. (10.1038/nrg2042) 17230199

[69] Brittle AL, Repiso A, Casal J, Lawrence PA, Strutt D. 2010 Four-jointed modulates growth and planar polarity by reducing the affinity of dachsous for fat. Curr. Biol. **20**, 803-810. (10.1016/j.cub.2010.03.056) 20434337 PMC2958304

[70] Bilousov OO, Kozeretska IA, Katanaev VL. 2012 Role of the gene Miniature in *Drosophila* wing maturation. Genesis **50**, 525-533. (10.1002/dvg.22016) 22290933

[71] Liu F, Li K, Li J, Hu D, Zhao J, He Y, Zou Y, Feng Y, Hua H. 2015 Apterous A modulates wing size, bristle formation and patterning in *Nilaparvata lugens*. Sci. Rep. **5**, 10526. (10.1038/srep10526) 25995006 PMC4440214

[72] Kudla AM, Miranda X, Nijhout HF. 2022 Data from: The roles of growth regulation and appendage patterning genes in the morphogenesis of treehopper pronota. *Dryad Digital Repository*. (10.5061/dryad.8pk0p2npg) PMC9174728 35673859

[73] Kudla AM, Miranda X, Nijhout HF. 2022 The roles of growth regulation and appendage patterning genes in the morphogenesis of treehopper pronota. *FigShare*. (10.6084/m9.figshare.c.6011669) PMC9174728 35673859

